# Artificial Intelligence in Dental Education: Overview of Teaching, Assessment and Academic Performance Prediction

**DOI:** 10.1016/j.identj.2026.109733

**Published:** 2026-07-11

**Authors:** Supachai Chuenjitwongsa, Tanit Arunratanothai, Paak Rewthamrongsris, Piyamas Sumrejkanchanakij, Kanoknadda Tavedhikul, Lakshman P. Samaranayake, Thanaphum Osathanon

**Affiliations:** a Dental Education Unit, Office of Academic Affairs, Faculty of Dentistry, Chulalongkorn University, Bangkok, Thailand; b Department of Anatomy, Center of Excellence for Dental Stem Cell Biology, Faculty of Dentistry, Chulalongkorn University, Bangkok, Thailand; c Department of Periodontology, Faculty of Dentistry, Chulalongkorn University, Bangkok, Thailand; d Center of Artificial Intelligence and Innovation, Faculty of Dentistry, Chulalongkorn University, Bangkok, Thailand; e Global Research Cell, Dr. D. Y. Patil Dental College and Hospital, Dr. D. Y. Patil Vidyapeeth, Pune, India; f Faculty of Dentistry, The University of Hong Kong, SAR China

**Keywords:** Artificial Intelligence, Pedagogy, Dental Education, Health Professions Education

## Abstract

Artificial Intelligence (AI) is increasingly used in dental education. The present study aimed to examine the current landscape of AI applications in dental education, focusing on their impact on teaching, assessment, and the prediction of academic performance. A literature search was performed based on articles published between November 2014 and March 2026 in the PubMed and Scopus databases. Studies focusing on AI applications in dental education were analysed across the domains: teaching and learning, assessment, and academic performance prediction. Forty-two publications met the inclusion criteria and were used as the evidential basis for this study. AI integration emphasised fundamental knowledge, use cases, and evaluation in teaching and learning. AI tools such as chatbots, simulations, and generative models enhanced student engagement and learning efficiency while supporting clinical decision-making. AI-assisted tasks, including radiograph interpretation and scientific writing, demonstrated improved outcomes. For assessment, AI showed moderate to high correlations with human evaluators for essay grading and thematic analysis. Further, AI models could forecast student outcomes, supporting personalised learning strategies and early intervention. AI applications have a vast potential to enhance dental education, particularly in improving teaching efficiency, personalised learning, self-directed learning, and assessment accuracy. Curriculum revisions and faculty training are necessary to fully integrate AI responsibly, leveraging its capabilities while maintaining the critical role of human oversight in education.

## Introduction

Artificial Intelligence (AI) has been rapidly transforming dentistry, offering significant advancements in diagnosis, treatment planning, predicting disease progression and outcomes, and patient education.^1^, ^2^, ^3^, ^4^, ^5^ An overview of different AI subclasses and their potential use in dentistry was previously reviewed.^5^^,^^6^ A recent FDI white paper on Artificial Intelligence for Dentistry, along with several other key publications, has highlighted the profound impact of AI on dental education and workforce development.^6^^,^^7^ AI can reshape traditional dental education methodologies and curricular scope, in particular by expanding simulation-based learning approaches. Furthermore, the utilisation of AI could facilitate the implementation of more learner-centric, constructivist pedagogical and educational designs that can adapt to individual student needs and learning styles.^8^ Given the rapid development of AI and its integration into dental care, dental professionals must acquire the skills to comprehend and utilise these tools effectively and ethically. In dental education, AI has the potential to enhance teaching and evaluation methods in both undergraduate and postgraduate dental curricula, offering new opportunities for personalised learning.^6^^,^^8^

Various aspects of the utilisation of AI in medical education have been explored, such as curriculum design, teaching tools, and assessment approaches.^9^^,^^10^ One notable application is the use of the large language model in objective structured clinical examinations (OSCEs) during the process of case development, standardisation, evaluation rubrics, and assessment procedures.^11^ Such applications of AI in dental education can significantly reduce time, cost, and workload for educators involved in OSCE preparation. Additionally, in the clinical arena, machine learning algorithms can assess and classify students into different levels of expertise based on their surgical skills performed in virtual reality simulations.^12^ This capability provides formative feedback for learners, enabling them to identify areas for improvement.

These examples clearly highlight the potential applications of AI in health education. However, the use of AI in dental pedagogy and education, particularly in teaching, assessment, and academic performance prediction, appears to be limited to date. This study examines the current landscape of AI applications in dental education, focusing on their impact on teaching and assessment.

## Methodology

A comprehensive literature review was conducted to gather core data on the use of AI in dental education, published between November 3, 2014 and March 10, 2026. The PubMed and Scopus databases were searched using the defined search term (Supplementary data 1). The identified articles were reviewed to extract the essential AI concepts, applications, and competencies relevant to dental education. The inclusion criteria for evaluation were research articles focused on the use of AI in dental education, specifically on teaching and assessment. The editorial comments, opinions, and reviews were excluded. In addition, studies that focused solely on AI's performance, on surveys of knowledge or clinical tasks without clear involvement or application in teaching and assessment, or on attitudes and perceptions were also excluded. The screening of the identified records was performed by T.A. and P.R. In the event of disagreement in the evaluation of the records, T.O. provided a third opinion, and a discussion was held to ensure agreement. Data extraction was conducted by T.O. and reviewed by P.R. and T.A.

## Results

**Fig. 1:**
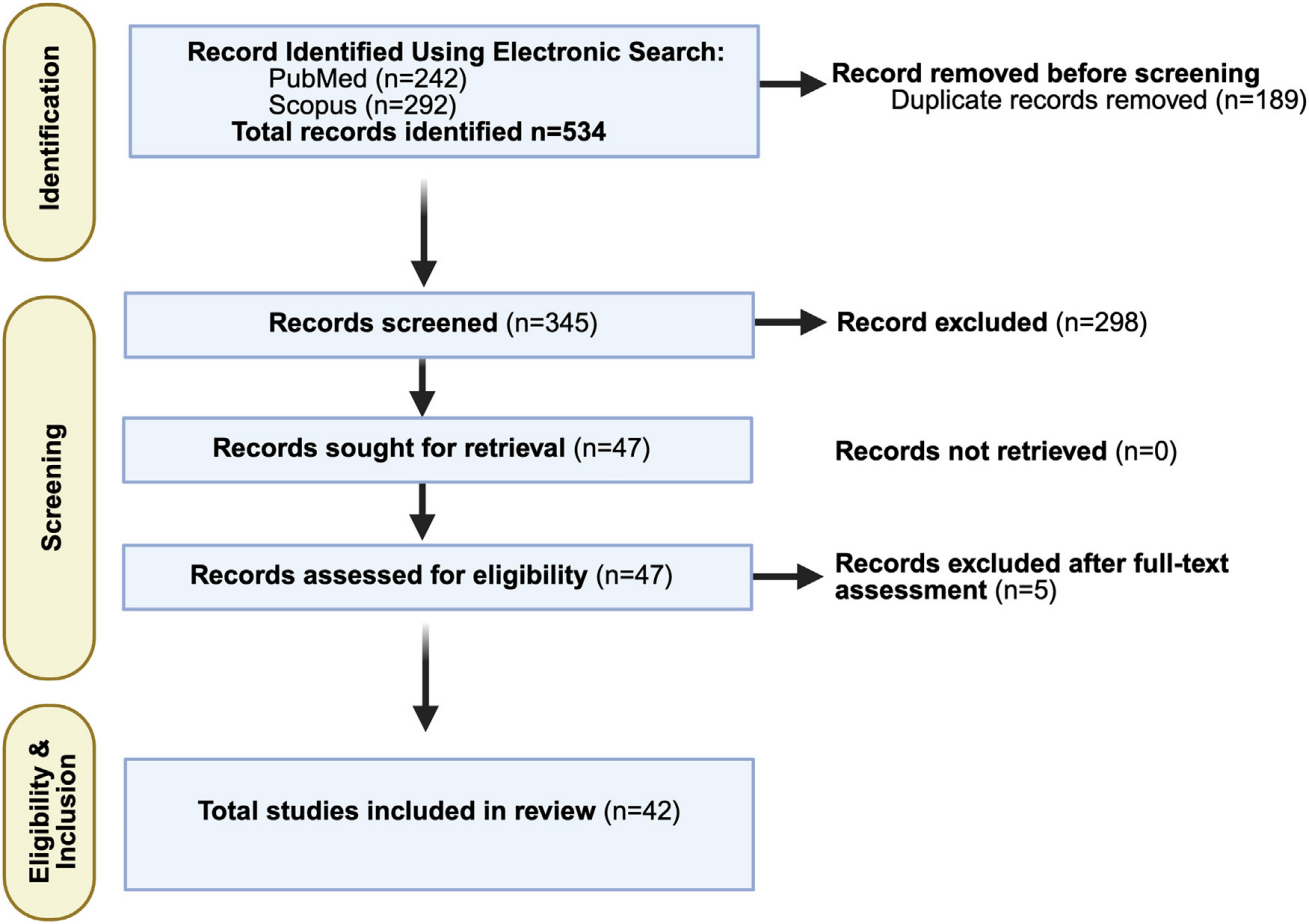
PRISMA flow diagram for the included studies. Created in BioRender. Osathanon, T. (2026) https://BioRender.com/px3qz2z.

The database search identified 534 records, including 242 from PubMed and 292 from Scopus. After removing 189 duplicate records, 345 unique articles remained for title and abstract screening. Following the initial screening and subsequent full-text eligibility assessment, 47 studies were further included for full-text screening. Following full-text assessment, five studies were excluded because they did not examine the application of AI within the context of dental education or were limited to the development of AI tools without incorporating evaluative components relevant to dental education. Hence, 42 studies met the inclusion criteria and were included in the final review^13^, ^14^, ^15^, ^16^, ^17^, ^18^, ^19^, ^20^, ^21^, ^22^, ^23^, ^24^, ^25^, ^26^, ^27^, ^28^, ^29^, ^30^, ^31^, ^32^, ^33^, ^34^, ^35^, ^36^, ^37^, ^38^, ^39^, ^40^, ^41^, ^42^, ^43^, ^44^, ^45^, ^46^, ^47^, ^48^, ^49^, ^50^, ^51^, ^52^, ^53^, ^54^, ^55^, ^56^, ^57^, ^58^, ^59^ (Figure 1).

The synthesis of publications highlighted the key areas that emerged from the review, leading to the categorisation of the studies into four domains: (1) teaching and learning (Table 1), (2) assessment (Table 2), and (3) performance prediction (Table 3).

**Table 1:** Summary of included studies regarding the AI use cases in teaching and learning for dental students.

Aspects in dental education	Educational level	Type of AI	Finding	References
Radiographic diagnosis	Undergraduate dental students	AI-assisted diagnostic software	Student training with AI software did not impact student ability to detect proximal enamel caries but improving the positive agreement of caries detection.	Schropp L, et al.^14^
Radiographic interpretation	Undergraduate dental students (3rd year)	not indicated	AI assistance significantly improved time for full-mouth radiograph series mounting; however, the accuracy was lower in the AI-assisted mounting.	Chang J, et al.^29^
Radiographic caries detection	Faculty / Dental instructors	not indicated (Second Opinion software)	AI tool (Second Opinion) assisted interpretation did not significantly improved diagnostic performance of instructors but increased the rate of unanimous agreement of sound surface and early-to-moderate dentinal caries.	Ji S, et al.^31^
Radiographic caries detection	Undergraduate dental students (4th and 5th year)	Convolutional neural network - Commercial AI caries detection software	AI assistance significantly improved proximal caries detection accuracy in both 4th- and 5th-year students.	Batgerel O, et al.^51^
Radiographic caries detection	Undergraduate dental students (1st and 3rd year)	not indicated - Overjet AI (commercial)	Radiographic caries detection ability improved by 43% to 46% after students completed the Overjet training module.	Caldwell J, et al.^57^
Orthodontic cephalometric training	Undergraduate dental students	not indicated - Digital AI-assisted cephalometric software	Digital and AI-assisted cephalometric training significantly enhanced landmarking accuracy in undergraduate students.	Lin J, et al.^48^
Orthodontic cephalometric tracing	Undergraduate dental students	not indicated (Munich Cephalometric Application for Training)	Students who used AI cephalometric teaching tool (MCAT) had lower variability in tracing.	Sabbagh H, et al.^46^
Anatomical landmark identification	Undergraduate dental students (3rd year)	Generative AI (LLM) ChatGPT-4	LLM-assisted group achieved better landmark identification accuracy than self-directed learning group. However, manual tracing achieved significant higher score than LLM-assisted groups.	Veerabhadrappa SK, et al.^28^
Radiology study assignment	Undergraduate dental students	Generative AI (LLM) ChatGPT-3.5 and ChatGPT-4.0	Students using ChatGPT in learning assignment had higher scores that those used traditional literature research strategy.	Kavadella A, et al.^45^
Radiographic education	Undergraduate dental students / Faculty	Generative AI (Generative Adversarial Network)	GAN-generated synthetic panoramic radiographs were radiologically similar to real ones and could be utilized as teaching tools without relying on patient-related data.	Schoenhof R, et al.^24^
Learning outcomes in pharmacology	Undergraduate dental students	Generative AI (LLM) ChatGPT-3.5	Students using ChatGPTto learn about side effects of drug used in dental practice showed improved pharmacology quiz scores compared to neither use ChatGPT nor read the description group.	Roganovic J^20^
Terminology learning (oral anatomy)	Undergraduate dental students (Year 1 and 2)	Generative AI (LLM) ChatGPT-4	ChatGPT-assisted learning significantly improved terminology comprehension and student engagement in learning was increased.	Rath A^52^
Emergency dental trauma management	Undergraduate dental students	Generative AI (LLM) ChatGPT-4o	Students performance on examination that allowing using ChatGPT-4.0 exhibited the accuracy similar to textbook and AcciDent application. However, students required less time when using ChatGPT4.0 and mobile application in answering the questions than those used textbook.	Haupt F, et al.^44^
Research Method course teaching	Postgraduate dental students	Generative AI ChatGPT	Blended, peer-led, AI-integrated research methods curriculum slightly improved academic performance compared to traditional lecture-based approach.	Natto ZS^49^
Academic task performance	Undergraduate dental students	Generative AI (LLM) ChatGPT	Utilisation of ChatGPT in scientific writing negatively influenced quality of the work compared to conventional literature search methods, despite the perception of students that indicated usefulness of ChatGPT in the tasks.	Saravia-Rojas MA^63^
Endodontic diagnosis	Undergraduate dental students (2nd year)	AI chatbot	No significant difference in post-test scores between chatbot and lecture groups; however, the students' perception was positive for chatbot in term of simplicity and fun.	Coelho MS, et al.^32^
Clinical skills (patient history taking)	Undergraduate dental students	Generative AI chatbot ChatGPT-3.5	AI chatbot enhanced student participations than clinical educator acting as patient.	Or Aj, et al.^39^
Clinical skills (patient history taking)	Undergraduate dental students (1st and 2nd year DDM)	Generative AI chatbot ChatGPT-4	Students exhibited higher competence in patient history-taking after using chatbot and showed positive motivations and enjoyment using AI chatbot for patient history-taking practice.	Or A, et al.^36^
Clinical simulation	Undergraduate dental students (2nd and final year)	Generative AI chatbot ChatGPT-4	AI role-play activity promoted peer learning and facilitated peer discussion. Students was engaging and perceived AI role play activity as authentic and clinical relevance.	Jones B, et al.^18^
Clinical simulation (virtual patient)	Undergraduate dental students (4th and 5th year)	Generative AI (LLM) GPT-4-turbo	AI virtual patient e-learning improved self-perceived diagnostic skills of the students.	Prinz M, et al.^27^
Clinical learning environment in implant clinic	Undergraduate dental students (predoctoral)	AI chatbot / Natural language processing	Students using custom-developed chatbot had the improvement of timeliness, interaction, and receptiveness while reduced anxiety compared with traditional Blackboard online platform.	Fang Q, et al.^25^
Clinical case simulation for temporomandibular disorders and orofacial pain	Undergraduate dental students	Generative AI (LLM) ChatGPT-3.5	AI simulations were comparable to standardised real patient interactions for TMD education. Students exposed to AI case simulation has less follow-up questions.	Rodrigues-Pereira P, et al.^34^
Clinical skills education	Undergraduate dental/medical students	Generative AI (LLM) ChatGPT-3.5	Students with ChatGPT integration in training had better skills performance on desktop virtual reality with high levels of self-efficacy and learning motivation but less cognitive load.	Huang S, et al.^56^
Personalized learning	Undergraduate dental students (Year 3)	Generative AI (LLM) Google Gemini	AI (Gemini)-personalized learning significantly improved formative assessment scores but not summative assessment scores. A strong positive correlation was observed between Gemini-usage and student performance. Students indicated that Gemini increased engagement.	Rath A^35^
Flipped classroom design (podcast generation)	Undergraduate dental students (clinical year)	NotebookLM	AI-enhanced flipped-classroom seminars showed positive perceived usefulness and engagement and students valued AI-generated podcast preparatory materials.	Eggmann F, et al.^54^

**Table 2:** Summary of included studies regarding the AI use cases in assessment for dental students.

Aspects in dental education	Educational level	Type of AI	Finding	References
Assessment (MCQ quality evaluation) -Oral Diagnosis and Radiology course	Undergraduate dental students (5th year)	Generative AI (LLM) ChatGPT-4	ChatGPT-generated MCQs had comparable discriminative index and item difficulty compared to educator-generated items.	Özer NE, et al.^21^
Assessment (MCQ generation and quality) - Oral and maxillofacial radiology course	Oral radiologists	Generative AI (LLM) ChatGPT-4o	AI-generated MCQs showed acceptable efficiency but significant limitations in higher-order cognitive levels. Experts and AI-detection tools could not differentiate between Ai-generated and human-created questions.	Elkersh NM, et al.^17^
Assessment (MCQ generated question) Oral Medicine assessment	Undergraduate dental students	Generative AI (LLM) ChatGPT-3.5	Human-generated items showed higher average scores and greater variability and higher discrimination, a wider difficulty range, better alignment with respondent abilities, and consistent fit indices.	Kiyani A, et al.^53^
Assessment (MCQ generated examination papers)	Undergraduate dental students	Generative AI (LLM) ChatGPT-4	AI-generated periodontology examination papers had higher content coverage and higher total scores, but lower discrimination indices than human-generated examination. Spit-half reliability was comparable to human-generated examination.	Ma X, et al.^41^
Assessment (examination question generation) National licence board examination	Undergraduate dental students (senior)	Generative AI (LLM) ChatGPT-4o	LLM-generated dental board-style questions met acceptable quality standards via item analysis; comparable difficulty and discrimination to human-expert questions.	Kim HS, et al.^22^
Root canal filling quality evaluation	Undergraduate dental students (preclinical setting)	Deep learning - RCFLA-YOLO (YOLOv11)	YOLOv11m achieved high performance for automated root canal filling length assessment, potential to use in assessment for preclinical dental education.	Ayhan M, et al.^43^
Endodontic training evaluation	Undergraduate dental students	Generative AI (LLM) ChatGPT-4o	Moderate agreement between ChatGPT-4o and expert evaluation of root canal treatments was reported. Students rated AI-feedback moderate useful.	Alpay S, et al.^50^
Feedback for student assignment histology questions	Undergraduate dental students	Generative AI (LLM) ChatGPT-4	Students perceived AI feedback that there were no differences in understanding mistakes, promoting critical thinking, feedback comprehension, or relevance. However, Ai feedback has less empathetic. Experts’ evaluation indicate that AI feedback was superior to identify higher mistakes and suggest for improvement.	Jayawardena CK, et al.^19^
Feedback (radiographic diagnosis) formative feedback	Undergraduate dental students (5th year)	Generative AI (LLM) ChatGPT-4o	AI-generated MeSH-based personalized formative feedback significantly improved post-test score and final test scores compared to standard correct/incorrect feedback at 1-month follow-up. Students indicated the AI-personalized feedback was more understandable, beneficial, and motivating than traditional feedback.	Gokkurt Yilmaz BN, et al.^40^
Automated essay scoring	Undergraduate dental students	Generative AI (LLM) ChatGPT-4	ChatGPT (GPT-4) demonstrated strong correlation with human assessors (ICC) for essay scoring; high reliability and agreement with human raters; potential for AI-assisted essay grading in dental examinations.	Quah B, et al.^42^
Automated grading open-ended clinical questions Periodontology course	Undergraduate dental students	Generative AI (LLM) ChatGPT-4 and DeepSeek-3	Deepseek-3 match human scores higher than ChatGPT-4 with less high-error rates. Both models over-scored on the incorrect results.	Hassanein FEA, et al.^47^
Essay grading assesses their clinical reasoning skills in the clinical dental sciences	Undergraduate dental students (final year)	Generative AI (LLM) ChatGPT-3.5	ChatGPT demonstrated ability to assess and grade formative essays using multiple prompt strategies.	Shamim MS, et al.^55^
Thematic analysis	Undergraduate dental students	Generative AI (LLM) ChatGPT	ChatGPT analysed themes and codes in comparable to those performed by evaluators.	Brondani M, et al.^26^
Skills evaluation	Undergraduate dental students (6th semester)	Machine learning	ML models provided valid and reliable evaluation of haptic skills (Class II amalgam and composite resin restorations). The grading is comparable to examiners.	Oguzhan A, et al.^38^
3D cavity preparation evaluation	Undergraduate dental students (preclinical)	Deep learning (neural network) / Generative AI ChatGPT	AI model for 3D cavity assessment had strong alignment with examiner scores.	El-Hakim M, et al.^59^

**Table 3:** Summary of included studies regarding the AI use cases in prediction for dental students.

Aspects in Dental Education	Educational Level	Type of AI	Finding	References
Academic performance prediction	Undergraduate dental students (pre-clinical)	Machine learning (logistic regression, random forest, decision tree, support vector machine)	ML models moderately predicted academic performance from pre-university cumulative grade point average.	Lestari W, et al.^30^
predicting skill levels	Undergraduate dental students / Dental professionals	Machine learning (Logistic Regression, Random Forest, Support Vector Machine (Linear RBF, Polynomial kernels), and a Neural Network)	AI-integrated dental handpiece provided real-time motion analysis could be used the information of deviation, take time and device type for skill level prediction.	Sallam M, et al.^58^

The majority of the included publications were focused on AI in teaching and learning. A summary of AI use cases in teaching and learning for dental students is presented in Table 1. Commonly reported use cases include AI-based chatbots for learning, AI-assisted radiographic interpretation, and AI-assisted academic writing. These AI-powered simulation tools offer students practical experience that enhances their learning efficacy.

AI use in dental education assessment was reported (Table 2), with fewer articles addressing the integration of AI into dental student evaluation. A study tested a large language model for grading esssays,^55^ revealing a correlation between AI assessments and human evaluations, with values ranging from moderate to high.^42^ In another study, instructors achieved an 85% accuracy rate in identifying whether reflective writing was generated by students or ChatGPT.^26^ Notably, ChatGPT performed thematic analysis of reflections similar to that of human raters, suggesting that assessors can differentiate between genuine student responses and AI-generated ones.^26^ This implies that AI tools could be leveraged to assist in thematic analysis during assessments. Overall, the large language model can support student self-assessment by providing formative feedback, thereby enhancing writing and learning outcomes. However, it is essential to note that AI should not be used as a stand-alone tool for summative evaluations.

The final domain focuses on predicting student performance (Table 3). A study evaluated several AI models to predict grades and forecast failures.^30^ Using pre-university cumulative grade point average (CGPA), the academic performance of pre-clinical dental students was predicted using AI models, including machine learning methods such as logistic regression, decision trees, random forests, and support vector machines. The results demonstrated that each model had unique predictive capabilities, with CGPA correlating with pre-clinical student performance, although the latter alone proved insufficient for accurate forecasting. Another study used an AI-integrated dental handpiece for real-time feedback, and the data can be used to predict students/ skill levels.^58^ These studies underscore the potential of AI as a predictive tool in dental education, which could prove useful in designing personalised learning strategies and goals for individual students.

## Discussion

**Fig. 2:**
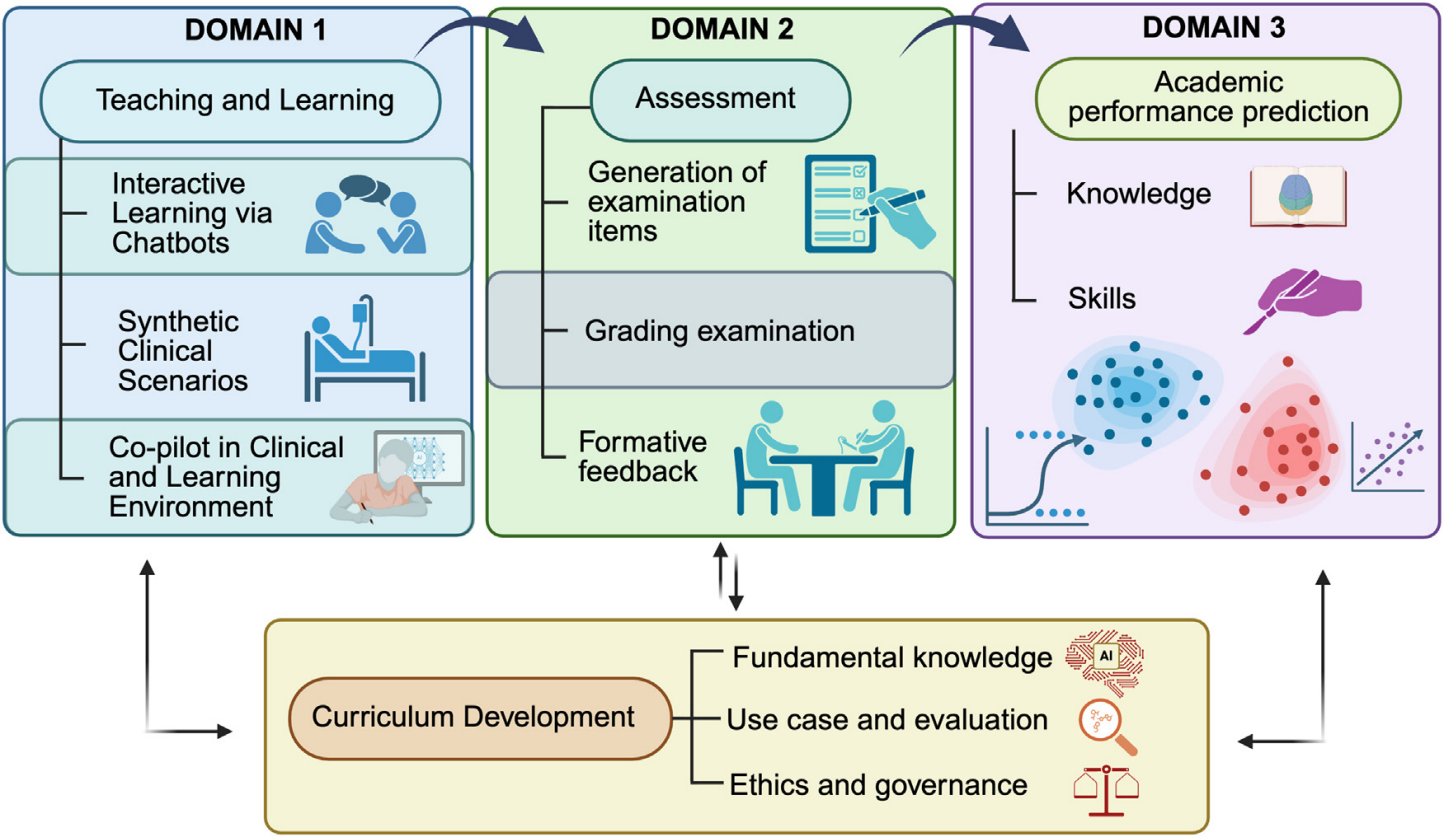
Schematic diagram of the AI in dental education, focused on teaching and learning, assessment, and academic performance domains. The proposed framework for curriculum development emphasises a knowledge foundation, AI use cases and evaluation, as well as ethics and governance. Created in BioRender. Osathanon, T. (2026) https://BioRender.com/px3qz2z.

The review outlined four key domains of AI integration in dental education: teaching and learning, assessment, performance prediction and curriculum development, which are discussed in some detail below Figure 2).

### AI in the Teaching and Learning Domain

In the teaching and learning domain, AI-powered interactive tools, such as chatbots and simulations, have shown promise in enhancing dental students' learning experience and engagement. With the rapid development of generative AI, students can use these tools to generate practice questions at their own pace or to acquire specific knowledge tailored to their learning needs.^60^

AI tools can also support the development of clinical skills. Pre-doctoral students’ perceptions of using AI chatbots in clinical education found that they improved interaction and engagement in a timely manner.^25^ Importantly, AI chatbots can also reduce faculty workload for student consultations.^25^ Using AI chatbots in a role-play learning environment improves peer discussion and peer learning.^18^ AI-powered tools can also support the development of students' interpersonal and communication skills. For example, AI-based conversational agents can simulate patient interactions, allowing students to practice their clinical skills. AI that mimics virtual patients has been employed to enhance clinical tasks for dental students, such as diagnostic skills, in which students engage in conversations with the chatbot until they reach the correct diagnosis. This approach has led to high levels of student satisfaction with the interactions.^61^ Moreover, AI chatbots can be specifically designed to improve patient history-taking skills, with students reporting their considerable value in honing clinical practice skills.^36^^,^^39^ However, concerns have been raised about the accuracy of the information provided, as well as issues related to time consumption and the potential for prompting knowledge.^25^^,^^60^ Furthermore, generative AI can be utilised to create clinical scenarios for specific practical purposes through interaction between humans and AI, for example, in patient education or patient communication settings. Virtual patients and AI-powered simulations can offer students opportunities to practice clinical skills and decision-making in a safe and controlled environment. In addition, generative AI models, such as generative adversarial networks, can be leveraged to create synthetic radiographic images. These images can serve as valuable tools, allowing students to practice interrogating a variety of clinical scenarios.^24^

A critical consideration in the development of AI tools for teaching and learning is the accuracy of the information they provide. Contemporary agentic AI systems can be designed to operate within constrained, predefined datasets, thereby reducing the likelihood of hallucinations. In this context, interactive AI systems that simulate clinical scenarios may offer substantial benefits for student practice and learning. Such an approach may serve as a viable alternative to standardised patients, whose use typically requires extensive training and greater financial investment.

Generative AI chatbots can also complement or simulate small group learning, a proven method in medical education that fosters collaboration, feedback, and critical thinking. These tools facilitate interactive learning and reinforce recall, deepening students’ understanding of lecture material and making them a versatile resource for medical education.^62^

One notable application of AI is through large language models, which have been explored as tools for scientific writing assignments. While students reported increased productivity using ChatGPT, the perceived usefulness of the scores for utilising evidence, evaluating arguments, and generating alternatives was higher with traditional search and writing methods.^63^ Another study reported that students using ChatGPT for their learning assignments had higher examination grades than those using a conventional literature search.^45^ Integration of student learning experience with Gemini resulted in an increase in assessment scores,^35^ as it seems to improve interactive learning, tailored and real-time feedback, and preparation for examinations. The contradictory findings regarding the benefits of large language models for writing assignments may be attributable to differences in students’ usage patterns. Large language models may be effective in identifying the central context of knowledge and suggesting relevant evaluative perspectives. However, summarisation and critical evaluation, when combined with human intellectual engagement, are likely to improve not only the quality of writing but also students’ internalisation of knowledge, thereby supporting the development of more coherent conceptual understanding. In some cases, large language models may also assist in refining language and enhancing readability, which may contribute to higher assessment scores. Taken together, students showed a high level of engagement and interest in interaction with ChatGPT as an alternative tool for information gathering.

Contradictory findings regarding the use of generative AI to support scientific writing were identified. One study reported that using ChatGPT in scientific writing adversely affected the quality of the work compared with conventional literature search methods, despite students’ perceptions that ChatGPT was beneficial for such tasks.^33^ Conversely, another study found that students who used ChatGPT for a learning assignment achieved higher scores than those who relied on traditional literature search strategies.^45^ It should be noted that these two studies examined different outcomes. One study focused on writing quality,^33^ whereas another examined knowledge retention.^45^ In other words, the latter study assessed learning associated with the use of ChatGPT during the literature review process. When using ChatGPT to locate relevant literature, students may refine and modify prompts to obtain the desired results. This iterative prompting process may contribute to the development of critical thinking and learning, thereby increasing the likelihood of knowledge construction when compared with traditional search methods.

Another approach to utilising AI tools in teaching and learning is the use of AI as a co-pilot. With this AI assistance, students performed specific tasks more efficiently. Students trained with AI exhibited a higher success rate in detecting proximal caries than those who were not trained with AI.^64^ Similar results were noted in the application of AI to student training for detecting cephalometric landmarks, where student-AI collaboration proved highly effective.^48^^,^^65^ Additionally, dental students using AI assistance completed full-mouth radiograph mounting faster than those performing the task manually, testifying to its effectiveness.^29^ However, this study also reported lower task accuracy in the AI-assisted group.^29^ In the same vein, Schropp et al. (2024) noted that using AI to assist in detecting proximal caries on bitewing radiographs did not significantly alter students’ ability to perform the task.^14^

The contradictory findings regarding the use of AI as a co-pilot in learning tasks may be attributable to several factors. First, a bias in favour of AI may foster overconfidence and excessive reliance, leading students to make judgments without sufficient reference to their existing knowledge. Accordingly, the role of human oversight in the use of AI should be emphasised within the dental curriculum to equip students with the competencies required for its appropriate and critical utilisation. Second, the effectiveness of AI may vary depending on students’ level of knowledge and how they engage with these tools. To use AI effectively as a co-pilot, students should possess a foundational understanding of the task. In such contexts, AI may reduce the time required to complete tasks by identifying points and supporting the construction of provisional knowledge scaffolds. However, optimal outcomes are more likely to be achieved when AI is used in conjunction with students’ own knowledge and judgment. Overall, the effective implementation of AI as a co-pilot in dental education requires careful instructional design that is appropriately aligned with students’ developmental stage and the nature of the learning task.

AI assistance can draw students' attention to specific areas of interest, potentially reducing the time required to perform particular clinical tasks. Nevertheless, it is crucial to emphasise the human role in teaching and learning when implementing this approach. With advancements in automated feedback, AI can create personalised learning experiences that foster critical thinking skills, thereby positively impacting student performance.^66^

Despite the considerable potential of AI in teaching and learning within dental education, it is important to recognise that a substantial component of dental training is centred on the psychomotor domain. Most studies have emphasised the use of AI to support cognitive development, particularly in relation to knowledge acquisition and critical thinking. The integration of AI into hand-skill training may address the current underrepresentation of psychomotor domain development in the literature. For example, AI–guided instruction could be incorporated into simulation laboratory settings to support students’ manual skill practice.

Despite its advantages, the use of AI should be closely monitored due to the potential for errors that could be ingrained in students' knowledge base. Hence, all AI systems must undergo rigorous evaluation and meet certified standards before release for general use. Moreover, AI tools used to answer questions should be regularly updated to ensure they provide up-to-date information, as guidelines for diagnosis and treatment in the medical and dental fields can change rapidly.

### AI in the Assessment Domain

Integrating AI into the assessment process can provide formative evaluations that can subsequently improve students' performance through a continuous virtuous cycle of learning. Generative AI can contribute to all aspects of OSCE preparation,^11^ including defining clinical skills to be assessed, creating case scenarios, developing questions and checklists, generating grading rubrics, and providing student feedback.

One study employed AI to create various types of assessment tests, such as A-type multiple-choice questions, integrated case-cluster multiple-choice questions, integrated short-answer questions, and objective structured practical examinations.^9^ While some AI-generated A-type multiple-choice questions exhibited issues such as item defects, multiple correct answers, and inappropriateness for the learner level, the overall quality of AI-generated tests generally demonstrated potential content validity.^9^ Systematic reviews have demonstrated that utilising AI to develop multiple-choice questions can improve accuracy, efficiency, and diversity. However, challenges remain in ensuring the validity, complexity, and reasoning ability of AI-generated multiple-choice questions.^13^

For integrated case cluster multiple-choice questions, clinical case studies can be appropriately tailored to different learner levels.^9^ For example, questions for pre-clerkship students focused on basic knowledge and skills (eg, 'know' and 'know-how' levels) while those for medical school graduate levels require a deeper level of interpretation of clinical information and decision-making for treatment approaches. AI can also create rubrics for grading and study guides for short-answer questions.^9^ By adjusting prompts, the case structures for these questions can be modified to generate responses that align with required criteria, such as difficulty level and context relevance.

Lastly, in objective structured practical examinations, AI-generated examinations have shown technical accuracy, effectively addressing core subtopics appropriate for the learner’s level.^9^ AI also generated explanatory notes that facilitate self-assessment and formative assessment. However, these AI-generated contributions necessitated careful oversight and input from educators to ensure quality, accuracy, validity and appropriateness.

A study comparing feedback given by humans and AI for histology questions revealed that the AI-generated feedback was often detailed, clear, and precise, aligning well with the marking scheme. Examples were sometimes provided to aid student understanding. This contrasted with human feedback, which included motivational components that acknowledged students' efforts. Despite the clarity of AI feedback, students rated the grading accuracy and the feedback quality of human assessment higher than that of AI.^19^ However, the study design did not blind students to the types of feedback provided, which may have introduced bias favouring human-generated responses.

When large language models were used for grading short-answer questions by medical students, strong correlations with human assessors were observed.^67^ Another study exploring deep learning for grading short-answer questions found that for simpler questions (eg, those worth 1 or 0 marks), deep learning AI exhibited high accuracy. However, its grading classification accuracy decreased for more complex questions.^68^ Interestingly, students perceived AI grading as less subjective than human marking.^68^ Nonetheless, AI is intended to serve as an assistant rather than a replacement for human assessors.^67^

The foregoing indicates that AI in the assessment domain can be used to generate questions, grade exams, and provide feedback, thereby considerably reducing educators' workload and offering personalised formative feedback to students. However, a mixed-methods study reported that dental educators expressed concerns regarding the effectiveness of assessments generated by AI for assessing student competency.^69^ Concerns persist regarding its accuracy in assessing practical skills, potential biases, and ethical implications.^70^ AI integration must be carefully managed to avoid unintended outcomes.^70^ Accordingly, the use of AI in summative assessment should be avoided. However, the periodic use of AI–based assessment for formative feedback may be beneficial in supporting the development of students’ knowledge and skills towards the attainment of the expected competencies. The findings suggest that AI can augment human assessment, highlighting the benefit of combining AI with traditional methods to enhance student evaluation.

### AI in the Student Performance Predicting Domain

Predicting student performance is a valuable resource of information that can assist both learners and educators in developing personalised educational plans to help students achieve their expected learning outcomes. Several studies have explored the potential use of AI, such as machine learning algorithms, in predicting student performance. For instance, artificial neural networks and Naive Bayes methods have been applied to predict undergraduate medical students' academic performance based on various factors such as demographics, family environment, socioeconomic status, prior educational experience, type of admission, and student progress.^71^ Diagnostic examination performance and sociodemographic characteristics have been found to be significant predictors of academic achievement.^71^

While AI-based prediction models leveraging pre-university CGPA data exhibited some correlation with student performance, they proved inadequate for accurately forecasting academic outcomes.^30^ Additionally, a natural language processing approach has also been used to develop predictive models for pre-clerkship student performance from narrative feedback.^72^ The effective predictive model included manually constructed topic groups, total word count of the narrative, and the number of below-expected ratings.^72^ Another study employed a total of 99 variables as inputs for predicting academic performance in medical students.^73^ These variables were categorised into demographic, quantitative, and qualitative factors. Among these, the national health sciences placement tests were the most powerful variable in predicting student performance. These predictive models enable educators to establish an early detection system to provide support to students, in good time, leading to the enhancement of the achievement of expected learning outcomes.

The example of a predictive model in medical education may not be fully applicable in dental education, as a major difference is the focus on the psychomotor domain. One study investigated an AI-integrated dental handpiece for collecting data and providing real-time feedback to students.^58^ These could be useful tools in the formative feedback process to improve hand skills. In addition, the AI can analyse data and identify predictive factors to determine students' skill levels.^58^ This approach should be further investigated and developed as a strategy for providing formative feedback and prediction to enhance students’ development in the psychomotor domain.

### AI in the Curriculum Development Domain

It was clear from the review of these core data that AI and its applications are expanding exponentially across various disciplines in dentistry. Hence, there is a pressing need to foster a new generation of dentists with AI literacy, necessitating the integration of AI literacy in dental curricula for both undergraduate and postgraduate programs.

In terms of curriculum development, learning outcomes related to oral and dental AI have been investigated and defined by expert groups, notably by the FDI World Dental Federation.^8^ The latter study reviewed various curricula and documents on AI literacy for medical professionals, employing expert interviews to scope and adapt the relevant items into the dental domain. The AI curriculum and the requisite learning outcomes were developed through an online Delphi consensus process, resulting in the identification of four key domains: fundamental knowledge, use cases, evaluation of medical and dental AI, and additional miscellaneous aspects.^8^

The first domain, fundamental knowledge, includes seven essential topics: (1) definitions and terms, (2) reasoning behind AI, (3) machine learning, (4) training, validation and testing, (5) reference tests, (6) dynamic and static AI, and (7) black box and explainability. The second domain addresses AI use cases in dentistry and various AI formulations applicable to specific contexts and typical dental setups. The third domain addresses the evaluation of medical and dental AI, encompassing topics such as evaluation matrices, interpretation, impact of AI applications on individual or community dental health outcomes, and examples of current AI applications. Lastly, the fourth domain emphasises the miscellaneous perspectives in AI usage, including generalisability and representativeness, explainability, autonomy and accountability, and governance. While these proposed learning outcomes cover the major areas related to AI integration in dentistry, they primarily emphasise knowledge acquisition. There appeared to be a lesser degree of focus on competencies and outcomes related to skill development and critical analytical thinking. To ensure that students develop evaluative thinking skills essential for understanding the rapid evolution of AI utilisation and application in dentistry, it is crucial to enhance the higher cognitive domains within the learning outcomes.

The dental curriculum should be dynamic and responsive to the rapidly evolving knowledge and technology in the arena of AI, which gained significant prominence in 2022. Hence, curriculum revision must aim not only to provide didactic knowledge in specific fields but also to equip students with the skills necessary to understand emerging innovations. Within the curriculum domain, the core component of AI literacy focuses on knowledge-level learning outcomes. However, enhancing higher cognitive and analytical capabilities, as well as technical competency related to AI use and application in dentistry, requires further attention due to the burgeoning developments in the AI arena.

In medical education, it has been proposed that the focus of AI in the curriculum should be on clinical use cases and the implications of AI in these contexts, rather than merely on the technical aspects of AI.^74^ However, some fundamental knowledge is crucial for a full understanding of its biases, limitations, and ethical concerns. For undergraduate medical students, the proposed competencies have been categorised by Triola & Rodman (2024) into six different domains: (1) fundamental knowledge of AI, (2) ethics and regulation, (3) application to clinical care and digital health, (4) assessing AI quality and accuracy, (5) data privacy and security, and finally (6) professionalism.^74^

The proposed AI competency domains in the dental curriculum are similar, focusing on fundamental knowledge, use cases, and the evaluation of medical and dental AI.^8^ However, data privacy and security are notably absent in the proposed competency domains in dental education.

Another study further defining the competency for AI in medical education has listed the following three competencies related to data management.^75^ In this regard, medical students should be competent in (1) maintaining healthcare records that AI can process, (2) recognising the significance of data collection, analysis, evaluation, and safety for developing AI applications in healthcare, and (3) properly analysing data obtained by AI in healthcare.

Integrating AI competencies into dental curricula faces several challenges, including the extensive content across basic and clinical disciplines, the complex curriculum revision process, and the lack of consensus on the required AI competencies. Additionally, there is a need to consider the vertical progression of AI competencies across different years of undergraduate studies, as well as between undergraduate and postgraduate levels. Hence, to prepare and equip students with the skills needed for the advanced AI era, the existing curriculum can be adapted by integrating AI-related content into case-based learning, seminars, and clinical settings.

## Limitations

The present study is subject to limitations related to its search terms and database selection. The literature search was conducted using only two databases, PubMed and Scopus, which may not have captured all potentially relevant articles. Nevertheless, these databases are widely recognised as principal sources for biomedical and interdisciplinary research, and using at least two databases is consistent with recommended practice to minimise the risk of overlooking eligible studies. In addition, the MeSH hierarchy term 'artificial intelligence' was employed in PubMed to encompass related sub-terms within the field. However, emerging terminology may not have been fully captured by this approach. Accordingly, future updated reviews should consider broader database coverage and more comprehensive search strategies to ensure a more complete identification of relevant publications.

## Conclusion

AI presents significant limitations; however, it heralds a transformative era in dental education. The integration of AI into teaching, learning, assessment, and performance prediction offers unprecedented opportunities to enhance educational outcomes. Based on the studies analysed in the present study, most articles are exploratory in nature. In addition, due to the small sample size and the single study institution, generalisation of AI use in dental teaching and assessment could not be achieved. Different curricula, learning styles, and cultures would impact the utilisation of AI in the dental curriculum. Hence, large-scale, multi-institutional studies with rigorous designs are indeed necessary to reveal and confirm the benefit of implementing AI in dental curriculum. Further, the journey toward fully incorporating AI into the dental curriculum will not be without its challenges. Educators must be equipped not only to harness AI's capabilities but also to navigate the ethical dilemmas and biases that may arise from its use.^76^^,^^77^ Lastly, it is crucial to emphasise the enduring role of human educators in this evolving landscape. Their expertise and judgment are vital in ensuring that AI serves as a complementary tool, enhancing rather than replacing the human elements of teaching and learning. As we continue to pursue advancements in AI technology, a balanced approach that prioritises both innovation and ethical considerations will be essential for fostering a future-ready dental education system.

## Availability of Data and Materials

Data sharing is not applicable to this article as no datasets were generated or analysed during the current study.

## Funding

The study is supported by the Faculty Development Fund, Faculty of Dentistry, Chulalongkorn University.

## Author Contributions

*Data interpretation*: Chuenjitwongsa, Arunratanothai, Rewthamrongsris, Sumrejkanchanakij, Samaranayake, Tavedhikul. *Critical revision of the manuscript*: Chuenjitwongsa, Arunratanothai, Rewthamrongsris, Sumrejkanchanakij, Samaranayake, Tavedhikul. *Conceptualising*: Osathanon. *Data acquisition*: Osathanon. *Interpretation*: Osathanon. *Manuscript drafting*: Osathanon.

## Declaration of Generative AI and AI-Assisted Technologies in the Writing Process

During the preparation of this work, the authors used AI tools to search for information, summarise the findings (Claude, Sonnet 4.6), create the draft (Claude, Sonnet 4.6), and improve readability and language (ChatGPT 5.4). After using this tool/service, the authors reviewed and edited the content as needed and took full responsibility for the publication's content.

## Conflict of Interest

None disclosed.

## Appendix Supplementary materials

Image, application 1

## Footnotes

Supplementary material associated with this article can be found in the online version at doi:10.1016/j.identj.2026.109733.

## References

[1] Achararit P., Manaspon C., Jongwannasiri C., Phattarataratip E., Osathanon T., Sappayatosok K. (2023). Artificial intelligence-based diagnosis of oral lichen planus using deep convolutional neural networks. Eur J Dent.

[2] Chindanuruks T., Jindanil T., Cumpim C. (2025). Development and validation of a deep learning algorithm for the classification of the level of surgical difficulty in impacted mandibular third molar surgery. Int J Oral Maxillofac Surg.

[3] Rewthamrongsris P., Burapacheep J., Trachoo V., Porntaveetus T. (2025). Accuracy of large language models for infective endocarditis prophylaxis in dental procedures. Int Dent J.

[4] Trachoo V., Taetragool U., Pianchoopat P., Sukitporn-Udom C., Morakrant N., Warin K. (2025). Deep learning for predicting the difficulty level of removing the impacted mandibular third molar. Int Dent J.

[5] Samaranayake L., Tuygunov N., Schwendicke F. (2025). The transformative role of artificial intelligence in dentistry: a comprehensive overview. part 1: fundamentals of AI, and its contemporary applications in dentistry. Int Dent J.

[6] Tuygunov N., Samaranayake L., Khurshid Z. (2025). The transformative role of artificial intelligence in dentistry: a comprehensive overview part 2: the promise and perils, and the international dental federation communique. Int Dent J.

[7] Schwendicke F, Uribe S, Cheung W, Verma M, Linton J, Kim YJ. Artificial Intelligence for Dentistry. Available fromhttps://www.fdiworlddental.org/sites/default/files/2023-01/FDI%20ARTIFICIAL%20INTELLIGENCE%20WORKING%20GROUP%20WHITE%20PAPER_0.pdf Accessed 20 Feb 2025.

[8] Schwendicke F., Chaurasia A., Wiegand T. (2023). Artificial intelligence for oral and dental healthcare: core education curriculum. J Dent.

[9] Sridharan K., Sequeira R.P. (2024). Artificial intelligence and medical education: application in classroom instruction and student assessment using a pharmacology & therapeutics case study. BMC Med Educ.

[10] Crotty E., Singh A., Neligan N., Chamunyonga C., Edwards C. (2024). Artificial intelligence in medical imaging education: recommendations for undergraduate curriculum development. Radiogr (L).

[11] Misra S.M., Suresh S. (2024). Artificial intelligence and objective structured clinical examinations: using ChatGPT to revolutionize clinical skills assessment in medical education. J Med Educ Curric Dev.

[12] Winkler-Schwartz A., Yilmaz R., Mirchi N. (2019). Machine learning identification of surgical and operative factors associated with surgical expertise in virtual reality simulation. JAMA Netw Open.

[13] Ali F., Talat H. (2024). AI integration in MCQ development: assessing quality in medical education: a systematic review. Life Sci.

[14] Schropp L., Sorensen A.P.S., Devlin H., Matzen L.H. (2024). Use of artificial intelligence software in dental education: a study on assisted proximal caries assessment in bitewing radiographs. Eur J Dent Educ.

[15] Choi S., Choi J., Peters O.A., Peters C.I. (2023). Design of an interactive system for access cavity assessment: a novel feedback tool for preclinical endodontics. Eur J Dent Educ.

[16] Rampf S., Gehrig H., Möltner A., Fischer M.R., Schwendicke F., Huth K.C. (2024). Radiographical diagnostic competences of dental students using various feedback methods and integrating an artificial intelligence application-a randomized clinical trial. Eur J Dent Educ.

[17] Elkersh N.M., Saif N., Samir W., Hussein G.A., GH M. (2026). AI-generated MCQs for pre-doctoral oral and maxillofacial radiology: acceptable efficiency with cognitive-level limitations. Eur J Dent Educ.

[18] Jones B., Desu A., Honig C.D.F. (2026). Artificial intelligence chatbots as virtual patients in dental education: a constructivist approach to classroom implementation. Eur J Dent Educ.

[19] Jayawardena C.K., Gunathilake Y., Ihalagedara D. (2025). Dental students' learning experience: artificial intelligence vs human feedback on assignments. Int Dent J.

[20] Roganovic J. (2024). Familiarity with ChatGPT features modifies expectations and learning outcomes of dental students. Int Dent J.

[21] Özer N.E., Balcı Y., Bölükbaşı G., İlhan B., Güneri P. (2025). Examining the role of artificial intelligence in assessment: a comparative study of ChatGPT and educator-generated multiple-choice questions in a dental exam. Eur J Dent Educ.

[22] Kim H.S., Kim G.T. (2025). Can a large language model create acceptable dental board-style examination questions? A cross-sectional prospective study. J Dent Sci.

[23] Xu X., Liu S., Zhu L. (2025). Development and evaluation of a retrieval-augmented large language model framework for enhancing endodontic education. Int J Med Inf.

[24] Schoenhof R., Schoenhof R., Blumenstock G., Lethaus B., Hoefert S. (2024). Synthetic, non-person related panoramic radiographs created by generative adversarial networks in research, clinical, and teaching applications. J Dent.

[25] Fang Q., Reynaldi R., Araminta A.S. (2025). Artificial Intelligence (AI)-driven dental education: exploring the role of chatbots in a clinical learning environment. J Prosthet Dent.

[26] Brondani M., Alves C., Ribeiro C. (2024). Artificial intelligence, ChatGPT, and dental education: Implications for reflective assignments and qualitative research. J Dent Educ.

[27] Prinz M., Schäfer E., Bürklein S., Donnermeyer D. (2026). Endodontic diagnostics training in undergraduate dental education: an observational pilot study on AI-driven virtual patient e-learning. Int Endod J.

[28] Veerabhadrappa S.K., Vadivel J.K., Roodmal S.Y., Porntaveetus T., Marya A., Selvaraj S. (2026). Panoramic landmarks: comparing LLM-assisted, manual tracing, and self-directed learning in dental education. Int Dent J.

[29] Chang J., Bliss L., Angelov N., Glick A. (2024). Artificial intelligence-assisted full-mouth radiograph mounting in dental education. J Dent Educ.

[30] Lestari W., Abdullah A.S., Amin A.M.A., Nurfaridah S.C., Ismail A. (2024). Artificial intelligence to predict pre-clinical dental student academic performance based on pre-university results: a preliminary study. J Dent Educ.

[31] Ji S., Daly L., Shah K., Bhoopathi V., Mallya S.M. (2025). Artificial intelligence in dental education: a pilot study of caries detection accuracy and instructor agreement. J Dent Educ.

[32] Coelho M.S., Piva G.B., Vasconcelos R.A., Toia C.C., Santos Zambon L., Brenelli S. (2026). Chatbot versus lecture in the teaching of endodontic diagnosis for undergraduate students-a pilot study. J Dent Educ.

[33] Saravia-Rojas M., Camarena-Fonseca A.R., León-Manco R., Geng-Vivanco R. (2024). Artificial intelligence: ChatGPT as a disruptive didactic strategy in dental education. J Dent Educ.

[34] Rodrigues-Pereira P., Dias-Calças M.A.P., Moreira Mélo A. (2025). Generative artificial intelligence-driven clinical case simulation in temporomandibular disorder education: ChatGPT versus real patients. J Dent Educ.

[35] Rath A. (2026). Empowering future dentists: a comprehensive mixed-methods exploration of artificial intelligence in personalizing year 3 clinical dental practice education. J Dent Educ.

[36] Or A., Sukumar S., Ma A. (2025). Enhancing dental students' history-taking skills with a generative artificial intelligence chatbot. J Dent Educ.

[37] Prakash K., Prakash R. (2024). An artificial intelligence-based dental semantic search engine as a reliable tool for dental students and educators. J Dent Educ.

[38] Oguzhan A., Peskersoy C., Devrimci E.E., Kemaloglu H., Onder T.K. (2025). Implementation of machine learning models as a quantitative evaluation tool for preclinical studies in dental education. J Dent Educ.

[39] Or A.J., Sukumar S., Ritchie H.E., Sarrafpour B. (2024). Using artificial intelligence chatbots to improve patient history taking in dental education (pilot study). J Dent Educ.

[40] Gokkurt Yilmaz B.N., Ozbey F., Yilmaz B.E. (2025). Effect of artificial intelligence-assisted personalized feedback on radiographic diagnostic performance of dental students: a controlled study. BMC Med Educ.

[41] Ma X., Pan W., Yu X.N. (2025). Evaluating AI-generated examination papers in periodontology: a comparative study with human-designed counterparts. BMC Med Educ.

[42] Quah B., Zheng L., Sng T.J.H., Yong C.W., Islam I. (2024). Reliability of ChatGPT in automated essay scoring for dental undergraduate examinations. BMC Med Educ.

[43] Ayhan M., Kayadibi İ, Aykanat B. (2025). RCFLA-YOLO: a deep learning-driven framework for the automated assessment of root canal filling quality in periapical radiographs. BMC Med Educ.

[44] Haupt F., Rödig T., Liersch P. (2025). Evaluating ChatGPT-4o as an educational support tool for the emergency management of dental trauma: randomized controlled study among students. JMIR Med Educ.

[45] Kavadella A., Dias da Silva M.A., Kaklamanos E.G., Stamatopoulos V., Giannakopoulos K. (2024). Evaluation of ChatGPT's real-life implementation in undergraduate dental education: mixed methods study. JMIR Med Educ.

[46] Sabbagh H., Ribnishki T., Hötzel L., Bronk L.V., Khazaei Y., Wichelhaus A. (2025). An open-source, ai-supported teaching tool in orthodontic education-assessment of acceptance and effectiveness. J Dent Educ.

[47] Hassanein F.E.A., Hussein R.R., Ahmed Y., El-Guindy J., DE A., Abou-Bakr A. (2026). Calibration of AI large language models with human subject matter experts for grading of clinical short-answer responses in dental education. BMC Oral Health.

[48] Lin J., Liao Z., Dai J. (2025). Digital and artificial intelligence-assisted cephalometric training effectively enhanced students' landmarking accuracy in preclinical orthodontic education. BMC Oral Health.

[49] Natto Z.S. (2026). Blended peer-led research curriculum with AI integration improves postgraduate students' academic performance and satisfaction: a quasi-experimental mixed-methods study. BMC Med Educ.

[50] Alpay S., Darafarin Y., Dagdelen B., Al-Shammari S.M.M., Buyukbayram I.K. (2025). Comparison of ChatGPT-4o and expert evaluation in endodontic education: a cross-sectional pilot study. BMC Med Educ.

[51] Batgerel O.E., Akkitap M.P., Sasany R. (2025). Diagnostic competence of senior dental students in detecting caries on panoramic radiographs with and without artificial intelligence assistance: a cross-sectional studycaries detection on panoramic radiographs. BMC Med Educ.

[52] Rath A. (2025). Leveraging ChatGPT to support terminology learning in oral anatomy: a mixed-methods study among linguistically diverse dental students. BMC Med Educ.

[53] Kiyani A., Hanif F., Muhammad M. (2025). Benchmarking ChatGPT-generated multiple-choice questions against faculty-authored items in dental education. Sci Rep.

[54] Eggmann F., Connert T., Weiger R., Herzog J. (2026). Preliminary insights into dental student satisfaction in flippedclassroom seminars enhanced by artificial intelligence-generated podcasts: a comparative study. Int J Comput Dent.

[55] Shamim M.S., Zaidi S.J.A., Rehman A. (2024). The revival of essay-type questions in medical education: harnessing artificial intelligence and machine learning. J Coll Physicians Surg Pak.

[56] Huang S., Wen C., Bai X. (2025). Exploring the application capability of ChatGPT as an instructor in skills education for dental medical students: randomized controlled trial. J Med Internet Res.

[57] Caldwell J., Parekh K., Crowther B. (2025). Performance evaluation of AI-based caries detection technology and its educational training module: a dual-phase investigation. Front Dent Med.

[58] Sallam M., Salah Y., Osman Y., Hegazy A., Khatab E., Shalash O. (2025). Intelligent dental handpiece: real-time motion analysis for skill development. Sens (Basel).

[59] El-Hakim M., Khaled H., Fawzy A., Anthonappa R. (2025). Clinician-led development and feasibility of a neural network for assessing 3D dental cavity preparations assisted by conversational AI. Dent J (Basel).

[60] Birks S., Gray J., Darling-Pomranz C. (2025). Using artificial intelligence to provide a 'flipped assessment' approach to medical education learning opportunities. Med Teach.

[61] Suarez A., Adanero A., Diaz-Flores Garcia V., Freire Y., Algar J. (2022). Using a virtual patient via an artificial intelligence chatbot to develop dental students’ diagnostic skills. Int J Env Res Public Health.

[62] Gilson A., Safranek C.W., Huang T. (2023). How does ChatGPT perform on the United States Medical Licensing Examination (USMLE)? The implications of large language models for medical education and knowledge assessment. JMIR med educ.

[63] Saravia-Rojas M.A., Camarena-Fonseca A.R., Leon-Manco R., Geng-Vivanco R. (2024). Artificial intelligence: ChatGPT as a disruptive didactic strategy in dental education. J Dent Educ.

[64] Ayan E., Bayraktar Y., Celik C., Ayhan B. (2024). Dental student application of artificial intelligence technology in detecting proximal caries lesions. J Dent Educ.

[65] Le V.N.T., Kang J., Oh I.S., Kim J.G., Yang Y.M., Lee D.W. (2022). Effectiveness of human-artificial intelligence collaboration in cephalometric landmark detection. J Pers Med.

[66] Mahrous A., Botsko D.L., Elgreatly A., Tsujimoto A., Qian F., Schneider G.B. (2023). The use of artificial intelligence and game-based learning in removable partial denture design: a comparative study. J Dent Educ.

[67] Morjaria L., Burns L., Bracken K. (2024). Examining the efficacy of ChatGPT in marking short-answer assessments in an undergraduate medical program. Int Med Educ.

[68] Hollis-Sando L., Pugh C., Franke K.B. (2023). Deep learning in the marking of medical student short answer question examinations: student perceptions and pilot accuracy assessment. Focus Health Prof Educ Multi-Prof J.

[69] Louca C., Tonni I., Leung A., Fine P. (2025). Artificial intelligence: Friend or foe in the assessment of dental students?. J Dent.

[70] Haruna-Cooper L., Rashid M.A. (2023). GPT-4: the future of artificial intelligence in medical school assessments. J R Soc Med.

[71] Monteverde-Suarez D., Gonzalez-Flores P., Santos-Solorzano R. (2024). Predicting students' academic progress and related attributes in first-year medical students: an analysis with artificial neural networks and Naive Bayes. BMC Med Educ.

[72] Maimone C., Dolan B.M., Green M.M., Sanguino S.M., Garcia P.M., O'Brien C.L. (2023). Utilizing natural language processing of narrative feedback to develop a predictive model of pre-clerkship performance: lessons learned. Perspect Med Educ.

[73] van Heerden B., Aldrich C., du Plessis A. (2008). Predicting student performance using artificial neural network analysis. Med Educ.

[74] Triola M.M., Rodman A. (2025). Integrating generative artificial intelligence into medical education: curriculum, policy, and governance strategies. Acad Med.

[75] Caliskan S.A., Demir K., Karaca O. (2022). Artificial intelligence in medical education curriculum: an e-Delphi study for competencies. PLoS One.

[76] Schwendicke F., Blatz M., Uribe S. (2023).

[77] United Nations Educational S, Cultural O (2022).

