# Supplementary material for: Artificial Intelligence in Dental Education: Overview of Teaching, Assessment and Academic Performance Prediction
Source: Int Dent J. 2026 Jul 11;76(5):109733. doi: 10.1016/j.identj.2026.109733 (PMC13381989; doi:10.1016/j.identj.2026.109733)
Supplement: Supplementary file 1 [file mmc1.docx]

**Supplementary data 1:** The search terms

**PubMed Search Terms**

("artificial intelligence"[Text Word] OR "artificial intelligence"[MeSH Terms]) AND ("education, dental"[MeSH Terms] OR "education, dental, continuing"[MeSH Terms] OR "education, dental, graduate"[MeSH Terms] OR "dental education"[Text Word] OR "dental examination"[Text Word] OR "dental assessment"[Text Word])

**Scopus Search Terms**

( TITLE-ABS-KEY ( artificial intelligence ) AND TITLE-ABS-KEY ( dental education , dental examination , dental assessment ) )
